# Development and first biomechanical validation of a score to predict bone implant interface stability based on clinical qCT scans

**DOI:** 10.1038/s41598-021-82788-y

**Published:** 2021-02-08

**Authors:** Dirk Wähnert, Andre Frank, Johanna Ueberberg, Lukas F. Heilmann, Odile Sauzet, Michael J. Raschke, Dominic Gehweiler

**Affiliations:** 1 Department of Trauma, Hand, and Reconstructive Surgery, University Hospital Muenster, Albert-Schweitzer-Campus 1, Building W1, 48149 Muenster, Germany; 2 Department of Trauma and Orthopedic Surgery, Protestant Hospital of Bethel Foundation, University Hospital OWL of Bielefeld University, Campus Bielefeld-Bethel, Burgsteig 13, 33617 Bielefeld, Germany; 3 School of Public Health and Centre for Statistics, University Bielefeld, Universitätsstraße 25, 33615 Bielefeld, Germany; 4 AO Research Institute Davos, Clavadelerstraße 8, 7270 Davos, Switzerland

**Keywords:** Preclinical research, Translational research, Tissues

## Abstract

Sufficient implant anchoring in osteoporotic bone is one major challenge in trauma and orthopedic surgery. In these cases, preoperative planning of osteosynthesis is becoming increasingly important. This study presents the development and first biomechanical validation of a bone-implant-anchorage score based on clinical routine quantitative computer tomography (qCT) scans. 10 pairs of fresh frozen femora (mean age 77.4 years) underwent clinical qCT scans after placing 3 referential screws (for matching with the second scan). Afterwards, three 4.5 mm cortical screws (DePuy Synthes, Zuchwil, Switzerland) were placed in each distal femur in the dia-metaphyseal transition followed by the second CT scan. The femur was segmented using thresholding and its outer shape was visualized as a surface model. A 3D model of the cortex screw in STL format was used to model the screw surface precisely. For each femur, the 3 cortex screw models were exactly positioned at the locations previously determined using the second CT scan. The BMD value was calculated at the center of each triangle as an interpolation from the measured values at the three vertices (triangle corners) in the CT. Scores are based on the sum of all the triangles’ areas multiplied by their BMD values. Four different scores were calculated. A screw pull-out test was performed until loss of resistance. A quadratic model adequately describes the relation between all the scores and pull-out values. The square of the best score explains just fewer than 70% of the total variance of the pull-out values and the standardized residual which were approximately normally distributed. In addition, there was a significant correlation between this score and the peak pull-out force (*p* < 0.001). The coefficient of determination was 0.82. The presented score has the potential to improve preoperative planning by adding the mechanical to the anatomical dimension when planning screw placement.

## Introduction

Osteoporosis, and resulting osteoporotic fractures, is an emerging disease in orthopedics and trauma surgery units. It is estimated that approximately 21% of all women and 6% of men aged 50 to 84 in the five largest countries of the European Union and Sweden (EU5+) suffer from osteoporosis^1^. In 2010, 2.46 million new fractures due to osteoporosis were estimated for these countries, 67% of which occurred in women^1^. Furthermore, it is known that osteoporosis is associated with an increase in mortality. Studies show that about 30% of excess mortality is directly attributable to the fracture event^1^. In absolute terms, this means that in the EU5+ countries, approximately 34,000 deaths each year are caused by osteoporotic fractures^1^.

There is a consensus among surgeons treating osteoporotic fractures: they must be stabilized or replaced by a prosthesis to allow early mobilization with full weightbearing^2,3^. However, sufficient implant anchoring is increasingly challenging trauma and orthopedic surgeons because of poor bone quality. A pragmatic solution to this problem is the use of implant augmentation, a.e. bone cement. This increases the load bearing area and thus reduces peak forces at the bone-implant-interface, lowering the risk of implant cut-out or cut-through. In addition, anchoring of the implants is improved, reducing the likelihood of loosening and migration. However, the use of bone cement is associated with prolonged surgery time, as well as additional risks to the patient and increased health care costs. Another problem of augmentation is the identification of patients who benefit from this measure. Since reliable objective preoperative values are lacking, each surgeon must make the decision to employ augmentation on a case by case basis intraoperatively.

Therefore, the aim of this study was the development and a first biomechanical validation of a bone-implant-anchorage score based on clinical routine quantitative computer tomography (qCT) scans of the distal femur.

## Material and methods

### Ethical approval

The human specimens were used and dissected in this examination in accordance to and under permission of the „Gesetz über das Leichen-, Bestattungs- und Friedhofswesen (Bestattungsgesetz) des Landes Schleswig–Holstein vom 04.02.2005, Abschnitt II, § 9 (Leichenöffnung, anatomisch)“. In this case, it is allowed to dissect the bodies of the donators (Körperspender/in) for scientific and/or educational purposes. All donators gave their informed consent. The project was reviewed by the internal board of the anatomical institute.

### Bone samples

For this study, 10 pairs of fresh frozen femora were used (6 females, 4 males). The mean age was 77.4 years (range 60 to 93). Mean bone mineral density (BMD) in the femoral head measured from qCT was 230 mgCaHA/ccm (SD ± 44).

### Implants

In this study, two CT scans were obtained from each specimen, and, to easily match them, 3 referential screws were placed in each distal femur prior to the first scan. These screws were custom-made of pure titanium with a length of 12.5 mm and a diameter of 3 mm to minimize artifacts.

For biomechanical investigation, 60 4.5 mm cortical screws (DePuy Synthes, Zuchwil, Switzerland) made of titanium alloy with a length of 80 mm were used. Three screws were placed in each distal femur.

### Study algorithm

First, three marker screws were inserted into each distal femur: One at the medial and lateral dorsal condyle at the cartilage-bone junction, and two others in the intercondylar notch from anterior (left femora) and caudal (right femora), respectively.

**Figure 1:**
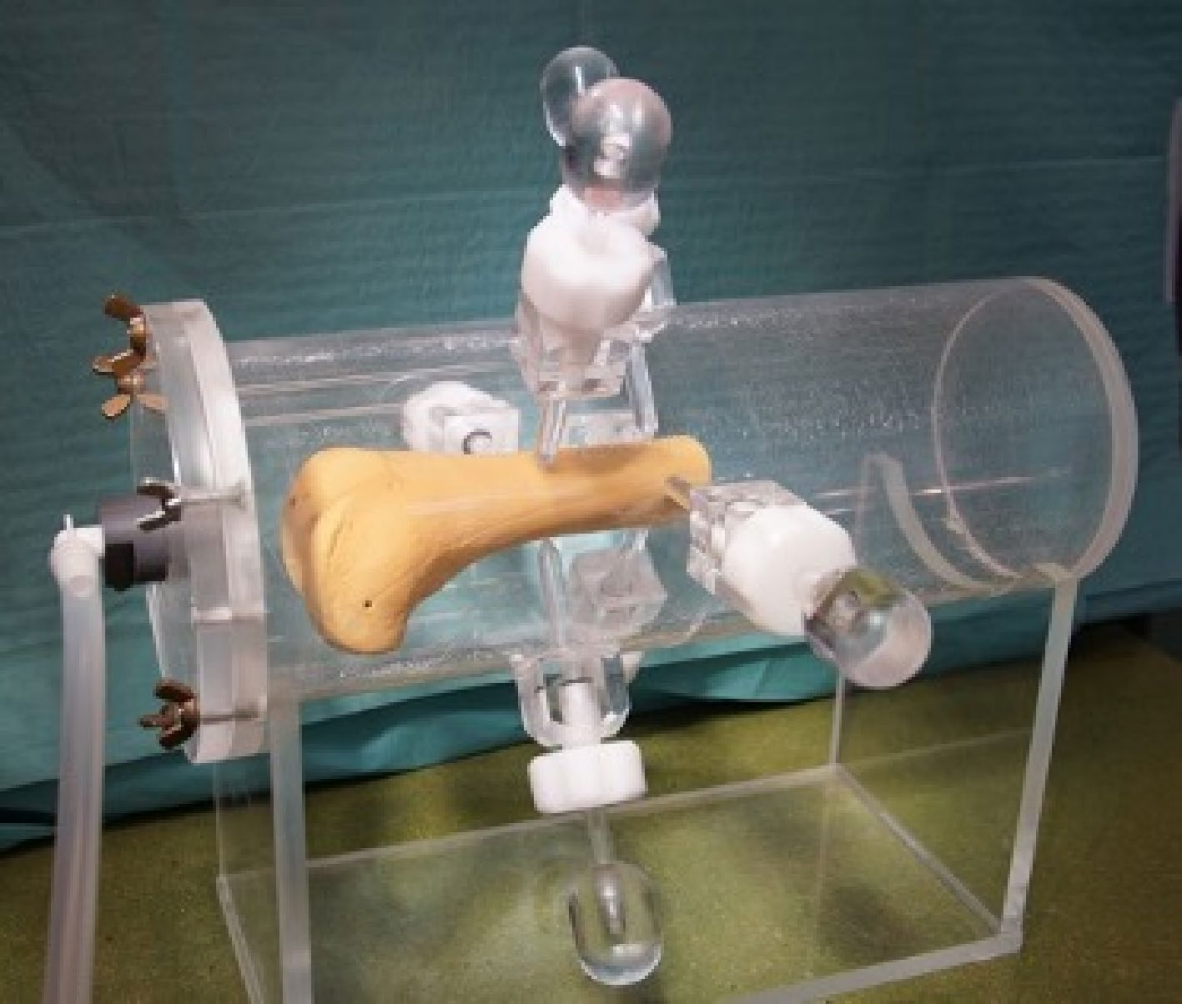
Picture of the custom made PMMA-cylinder with an artificial distal femur placed inside; for evacuation of air a negative pressure was applied. Afterwards the qCT was performed with the specimen inside the cylinder.

Before performing the qCT, the samples were thawed for 24 h at 8 °C. Then the clamping was done in a custom-made, water-filled PMMA cylinder (Fig. 1). The samples were held in the diaphyseal area with 3 pins. For evacuation of air, a negative pressure of 1 bar was applied for 10 min. The cylinder was then sealed and placed in the CT (Siemens Somatom Emotion 6, Siemens Health Care GmbH, Erlangen, Germany). A qCT scan was performed using the Siemens Osteo phantom with the following settings: 0.63 mm slice thickness, effective mAs of 80 at 110 kV, Pitch 1.8, reconstruction kernel B60s. After this scan, the specimens were frozen again.

**Figure 2:**
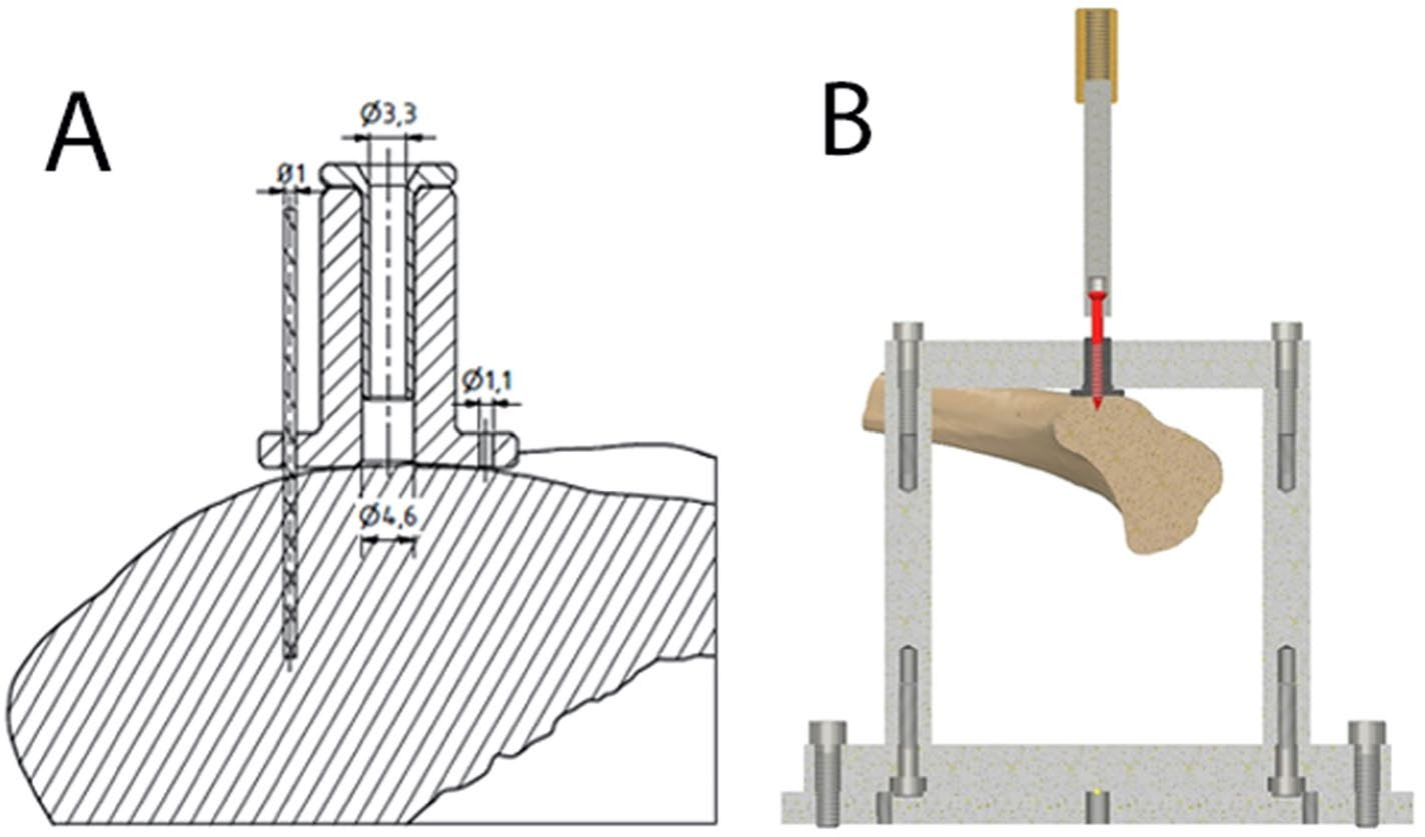
(**A**) Schematic drawing of the custom-made guiding device fixed with 1 mm K-wires to the distal femur allowing exact axial alignment of the screw. (**B**) Drawing showing pull-out of the 40 mm deep inserted screw in the custom-made jig.

In the next step, 3 cortical screws were placed in each distal femur, 2 lateral and 1 medial, each in the dia-metaphyseal transition. In preparation of this, the samples were thawed for 24 h at 8 °C again. Afterwards, the screws were inserted via a custom-made guiding device to ensure an exact axial alignment of the screw to the respective bone surface. This will later allow for a purely axial pull-out test without bending loads. First, the guiding device was fixed to the bone using three 1 mm K-wires (Fig. 2A). Next, the hole was drilled using a 3.2 mm drill bit. Screws were placed at a depth of about 40 mm into the bone (Fig. 2B). After insertion of the screws, the second computed tomography (Somatom Definition AS+, Siemens Health Care GmbH, Erlangen, Germany) was performed with identical parameters. Rigid registration of the second CT scan to the first was performed in Amira (Amira 6.5, FEI SAS a part of Thermo Fisher Scientific, Hillsboro, USA). Likewise, the exact positions of the cortical screws within the first CT scans were obtained by rigid registration of cortex screw models to the cortical screws in the second CT scan.

### Pull-out-test

The pull-out test was performed using a universal material testing machine (Zwick/Roell Z05, Zwick, Ulm, Germany), equipped with a custom-made jig, in which the screw head was attached to the machine actuator in a sleeve, and the distal femur was mounted freely under an counterhold (Fig. 2B). Due to the standardized screw placement, an axial pull-out load without bending load was possible. The screw pull-out test was performed in displacement-controlled mode with a crosshead speed of 25 mm/min. This pull-out test was performed until loss of resistance was present.

### BMD-score

**Figure 3:**
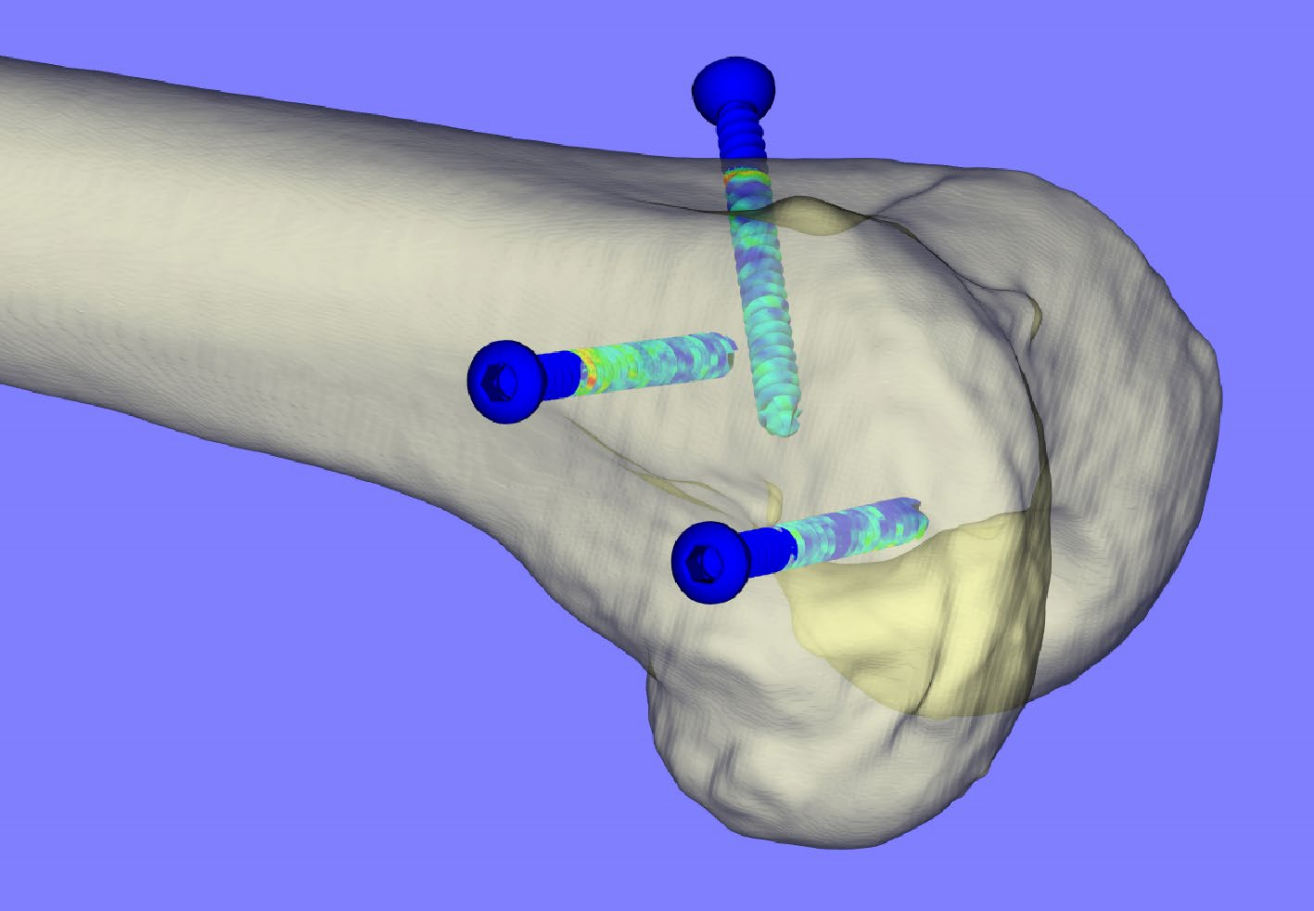
Exemplary image of a distal right femur as displayed in the software. The BMD value of triangles that lie outside the bone surface model is set to 0.

For the BMD score calculation, software was developed in the C++ programming language using the Visualization Toolkit (VTK)^4^. The femur was segmented using thresholding and its outer shape was visualized as a surface model. A 3D model of the cortex screw in STL format consisting of a triangular surface mesh of 272,064 triangles was used to model the screw surface precisely. For each femur, the 3 cortex screw models were exactly positioned at the locations previously determined using the second CT scan (Fig. 3).

**Figure 4:**
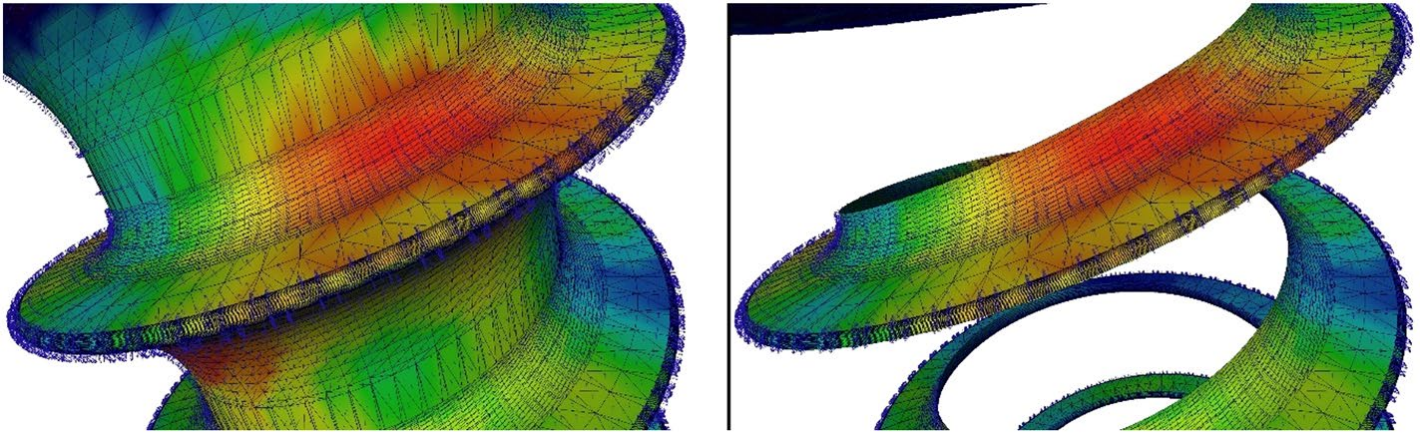
Enlarged view of an exemplary screw (the screw head would be above the image) with triangulation and triangle normals displayed. Left: complete screw; Right: triangles used for the weighted score.

**Figure 5:**
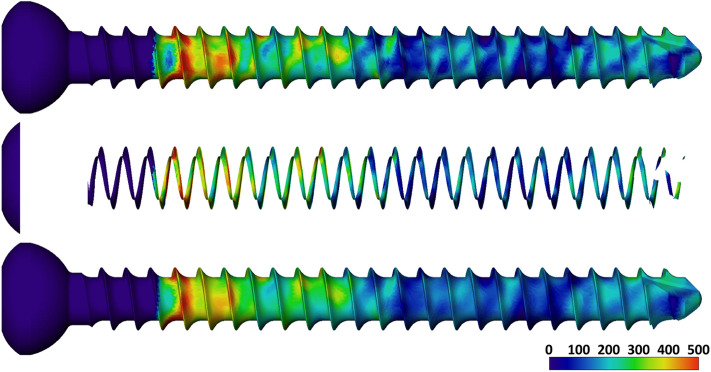
4.5 mm screw with bone mineral density projected onto the surface, Top: Score_0.0, Middle: weighted score, Bottom: Score_0.6.

The score for each individual screw was determined as follows. For each triangle of the screw surface mesh, determined by tree spatial coordinates each (three vertices), the triangle area, the BMD value, and the angle of the triangle normal to the screw axis (i.e. in which direction the triangle surface points, see Fig. 4) were calculated. The screw axis was defined pointing from the screw tip to the head. The BMD value was calculated at the center of each triangle as an interpolation from the measured values at the three vertices (triangle corners) in the CT. For all triangles that were outside the bone surface model, the BMD value was set to 0 (Figs. 3, 5). All negative BMD values ​​within the bone were also set to 0.

Scores are based on the sum of all the triangles’ areas multiplied by their BMD values. Four different scores were calculated. One score (Score_0.0, Fig. 5 Top) measures the local bone mineral density exactly at the spatial locations (vertices) of the triangles in the CT. Another score (Score_0.6, Fig. 5 Bottom) measures the bone density in a sphere with a radius of 0.6 mm averaged around the respective vertices. In addition, the triangles were weighted according to their angle (of the triangle normal to the screw axis). For these scores (Score 0.0w and Score 0.6w), only triangles with an angle smaller than 85° (Figs. 4, 5 Middle), were used and scored four times. A triangle with an angle of 0° would point with its surface in the direction of the screw head and thus counteract the pull-out to the maximum. The larger the angle becomes, the less it opposes the pull-out.

### Statistical evaluation

For statistical evaluation, Microsoft Excel 2016 (Version 16.31, Microsoft Cooperation, Redmond, USA) and STATA (Version 16, STATA Corp LLC, Texas, US) was used. The predictive value of the four scores for the pull-out values was carried out by means of a mixed-effects linear regression. Akaike´s information criterion (AIC) was used to identify which score best fit the pull-out data^5^. Significance was defined as *p* ≤ 0.05.

## Results

### BMD-scores

**Figure 6:**
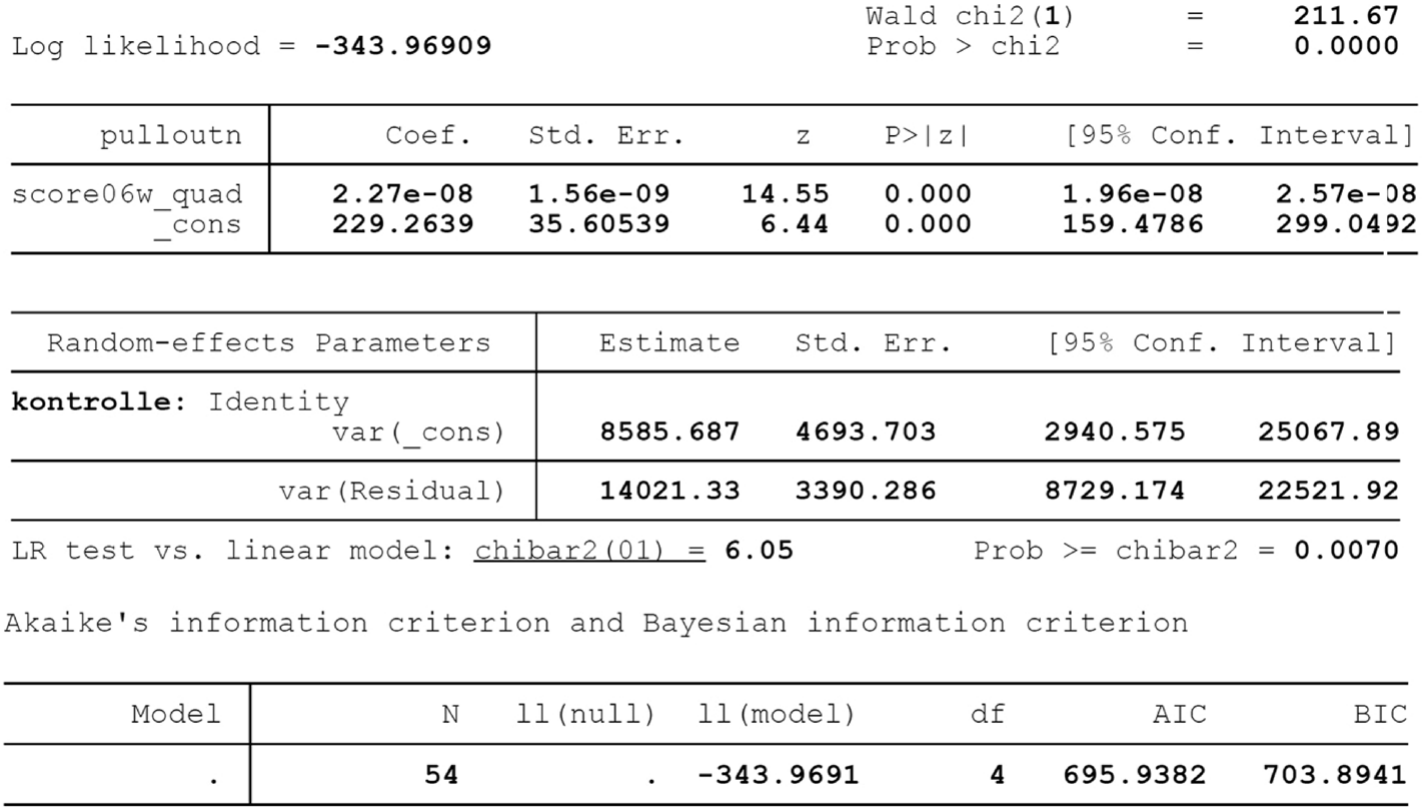
Statistical results of Regression Model for the Score0.6w.

A quadratic model adequately describes the relation between all the BMD scores and pull-out values. The lower AIC was found for the BMD score based on the weighted sum of the 0.6 mm sphere (Score_0.6w, Fig. 6). However, the differences in AIC were minimal.

### BMD-Score_0.6w and pull-out

There was a significant correlation of the calculated Score_0.6w and the peak pull-out force (*p* < 0.001). The coefficient of determination was 0.82.

**Figure 7:**
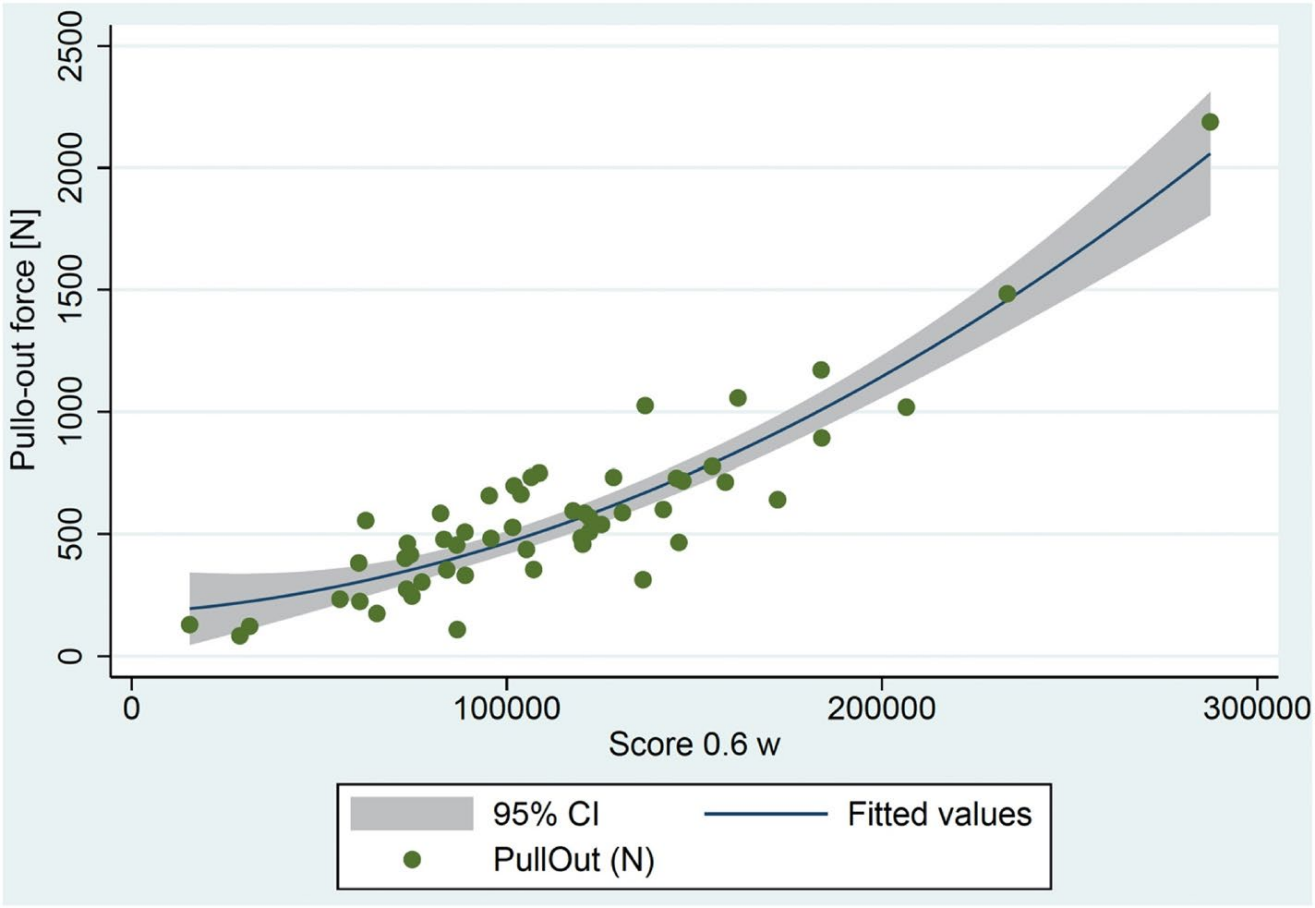
Diagram of the Score_0.6w including the quadratic regression and the 95% confidential interval.

The square of Score_0.6w explains just under 70% of the total variance of the pull-out values and the standardized residual, which were approximately normally distributed (Fig. 7).

## Discussion

This study investigates the bone implant interface stability based on a BMD score calculated from clinical qCT at the distal femur. This work was able to develop a score with a predictive value to the measured pull-out force of a 4.5 mm screw.

With the introduction of locking plates, the fracture fixation of osteoporotic fractures in particular has been significantly improved. This resulted, however, in failure at the bone-implant interface due to the reduced bone quality^6–8^. To overcome this problem, the screw augmentation has been developed to increase screw-bone contact and therefore increase the load bearing area^9^. Numerous biomechanical studies confirm the significant superiority of augmented osteosynthesis under cyclical loading in different anatomical regions^10–16^. Clinical studies thus far show no significant advantage of implant augmentation techniques. A prospective multicentre, randomized, patient-blinded trial investigated unstable pertrochanteric fractures (n = 223) treated with a standard proximal femoral nail either with (n = 105) or without (n = 118) additional augmentation. None of the patients with additional augmentation needed revision surgery due to mechanical failure or symptomatic implant migration, and 6 of the patients without augmentation required such revision; this difference was statistically not significant^17^.

The recently introduced variable angle stable locking system allows the surgeon to vary the screw angle and direction. Thus, a fracture adapted screw positioning can be performed with the benefit of angular stable locking^18^. Combining this with a preoperative plan regarding the screw position, an osteosynthesis that is modelled to the specific fracture and bone quality is possible. This might increase the mechanical stability of the bone implant construct and has the potential to decrease complication rates.

In the last years, preoperative planning using clinical CT scans, 3D printing, or virtual reality, as well as intraoperative navigation and 3D printed templates has become increasingly more routine, especially in complex cases^19–23^.

Using the presented method, preoperative planning of screw position can be used to predict screw bone interface stability and determine the construct stability and the failure risk. Therefore, preoperative planning can be used to optimize implant positioning in terms of biomechanical stability. Additionally, patients at high risk of bone implant interface failure can be identified. In these cases, additional techniques like implant augmentation or double plating of the fracture can be used to increase stability and reduce the failure rate. Even though no perfect prediction of screw stability is possible, this method presents the huge advantage that the score calculation can be performed almost in real time on any regular PC based only on a standard clinical CT. Therefore, it would be very easy to integrate this score calculation into conventional navigation systems respectively their software. Even if there is no BMD phantom available in the CT, a qualitative statement about the relative screw stability can still be made, and the location with the highest stability can always be determined.

It was not the aim of the study to optimize the score to the last for this special case. The choice of the two variants—weighting and averaging by means of the sphere—was rather based on the following considerations: In the case of weighting, the background consideration was that areas of the screw that do not act against the pull-out should theoretically have no influence on the pull-out resistance, and should therefore have no relevance for this score. When averaging the BMD value measurement with a sphere, the focus was on also being able to take into account CT data sets with a lower resolution, as the effect would be comparable.

This study also has limitations. The sample size is small. Especially in the extreme low and high BMD regions (very osteoporotic bone and very hard bone), the number of measurements is low and only one screw type was tested. Furthermore, the pure pull-out does not represent the clinical setting of failure. Nevertheless, this study was able to create a BMD score for an orthopaedic implant (screw) based on clinical qCT scans with a significant correlation to the pull-out values that can easily be calculated in real time.

The score published here in no way claims to replace a perfectly validated patient-specific FE model and all its capabilities. Rather, until all hurdles for a software based on a patient-specific FE model are overcome, it is meant to show a way how valuable supporting information could currently be provided to surgeons.

## Conclusion

The presented bone-implant-anchorage score calculated from clinical qCT correlates with the pull-out values. This technique can be used in preoperative planning to optimize implant positioning with the goal of improved anchorage and, in the future, to identify patients at risk for implant failure.
